# The New Hyperspectral Analysis Method for Distinguishing the Types of Heavy Metal Copper and Lead Pollution Elements

**DOI:** 10.3390/ijerph19137755

**Published:** 2022-06-24

**Authors:** Jianhong Zhang, Min Wang, Keming Yang, Yanru Li, Yaxing Li, Bing Wu, Qianqian Han

**Affiliations:** 1 College of Geoscience and Surveying Engineering, China University of Mining and Technology-Beijing, Beijing 100083, China; lenova20@163.com (J.Z.); lyr_98@163.com (Y.L.); yaxing_98@163.com (Y.L.); wubing9362@163.com (B.W.); 15540987968@163.com (Q.H.); 2 Youth League Committee, North China University of Science and Technology, Tangshan 063210, China; mwang2201@163.com

**Keywords:** spectral analysis, environmental heavy metal pollution, corn leaves, machine learning, multivariate empirical mode decomposition

## Abstract

In recent years, the problem of heavy metal pollution in agriculture caused by industrial development has been particularly prominent, directly affecting food and ecological environmental safety. Hyperspectral remote sensing technology has the advantages of high spectral resolution and nondestructive monitoring. The physiological and biochemical parameters of crops undergo similar changes under different heavy metal stresses. Therefore, it is a great challenge to explore the use of hyperspectral technology to distinguish the types of the heavy metal copper (Cu) and lead (Pb) elements. This is also a hot topic in the current research. In this study, several models are proposed to distinguish copper and lead elements by combining multivariate empirical mode decomposition (MEMD) transformation and machine learning. First, MEMD is introduced to decompose the original spectrum, which effectively removes the noise and highlights and magnifies the weak information of the spectrum. The successive projections algorithm (SPA), competitive adaptive reweighted sampling (CARS), and iteratively retaining informative variables (IRIV) were used to screen the characteristic bands and were combined with extreme learning machine (ELM), support vector machine (SVM), and general regression neural network (GRNN) algorithms to build models to distinguish the types of Cu and Pb elements. The quality of the model was evaluated using accuracy (*A*), precision (*P*), recall (*R*), and *F*-score. The results showed that the MEMD-SPA-SVM, MEMD-CARS-SVM, MEMD-SPA-ELM, MEMD-CARS-ELM, and MEMD-IRIV-ELM models intuitively and effectively distinguished the types of Cu and Pb elements. Their accuracy and *F*-scores were all greater than 0.8. To verify the superiority of these models, the same model was constructed based on first derivative (FD) and second derivative (SD) transformations, and the obtained classification and recognition accuracy (*A*) and *F*-score were both lower than 0.8, which further confirmed the superiority of the model established after MEMD transformation. The model proposed in this study has great potential for applying hyperspectral technology to distinguish the types of elements contaminated by Cu and Pb in crops.

## 1. Introduction

In this era, with the continuous acceleration of industrialization and urbanization [1], the scale of the development of production activities, such as mining, smelting, and various processing and manufacturing industries, has been at a relatively high level. Heavy metal pollution is a serious environmental problem. Among the many heavy metal pollutants, copper (Cu) and lead (Pb) pollution has attracted significant attention [2]. Copper is an essential element for plant growth. When plants contain a small amount of copper, it can promote plant growth, whereas excess copper can be harmful to plant growth, affecting plant photosynthesis, causing loss of green color, growth retardation and, in serious cases, leading to plant death [3]. Lead is harmful to plants, mainly by affecting photosynthesis and respiration. People in an environment with more serious lead pollution will experience damage to the human nervous system and blood circulatory system, which can lead to other diseases [4]. Therefore, heavy metal pollution monitoring and control technologies have become a popular research topic in the field of environmental protection. Heavy metal pollution in agriculture has attracted considerable attention [5]. Heavy metals migrate slowly in agricultural soils and accumulate mainly in areas where they are not easily decomposed. Heavy metals are transferred into the food chain through crops grown in polluted soil, which harm people or other organisms that feed on them. In serious cases, they endanger food safety and public health [6,7,8]. To achieve accurate crop management and environmental protection, it is necessary to monitor and distinguish the types of heavy metal pollutants in a timely and effective manner.

Traditional techniques for the investigation of heavy metal pollution, such as field sampling, indoor chemical analysis, and geostatistical interpolation, require large areas, extensive field sampling, and experimental analysis, which are time consuming, labor intensive, and costly [9]. Currently, gradually emerging hyperspectral remote sensing technology can achieve rapid, nondestructive, and large-area monitoring and is widely used in the exploration of agricultural heavy metal pollution screening and detection [10,11,12]. Early spectral analyses of the interaction between vegetation and heavy metals can be traced back to the 1970s and the 1980s. For example, Milton et al. [13] and Horler et al. [14] defined the position of the red edge, which proved that the red edge of the vegetation spectrum curve would be blue-shifted under heavy metal stress. Many researchers have used hyperspectral techniques to monitor heavy metal pollution in vegetation leaves [15] and whole plants [16]. The arsenic (As) concentration in soil can be estimated successfully by combining spectral preprocessing methods, such as Savitzky Golay smoothing (SG), first derivative (FD), and mean-centering (MC), and constructing an optimal model by measuring the spectra of growing rice plants [17]. Wavelet analysis has been applied to hyperspectral data processing and a monitoring model for heavy metal pollution in rice [18]. Zhang et al. [19] predicted the Cu content in corn leaves under Cu stress by developing a hyperspectral analysis model. The As content of ferns can be predicted by pot experiments using the first derivative (FD) of spectral reflectance [20]. The near-infrared (NIR) bands in spinach leaves under arsenic stress had a significant correlation with arsenic content [21]. Zhou et al. [22] used hyperspectral technology to detect Pb content in lettuce leaves and achieved good results. The wavelet transform of the spectrum combined with least squares support vector machine regression (WT-LSSVR) can effectively detect cadmium residues in tomato leaves [23]. Spectral reflectance of plant leaves changes under heavy metal stress. Several studies have found that relative chlorophyll content is significantly correlated with the content of heavy metals in leaves. It can be seen that the combination of hyperspectral and chlorophyll content detection can also be used for the diagnosis of heavy metal pollution [24]. Establishing a correlation model between spectral indices and Cd content in crop tissues can also achieve rapid quantitative analysis of Cd pollution, thereby rapidly determining vegetable quality [25,26]. A new hyperspectral vegetation index (CSVI) was found to be satisfactorily correlated with Cd content in plant leaves [27].

In summary, previous studies on the inversion of heavy metal pollution using hyperspectral techniques have focused on the prediction of heavy metal content. In contrast, there are few studies on the identification of heavy metal elements, which are relatively rare. Given this deficiency, the identification of heavy metal elements was performed in this study. The analysis of spectral data using the spectral analysis of signals is a new method that can decompose spectral data into multiple layers to fully explore the spectral features at different frequencies. Multivariate empirical mode decomposition (MEMD) is a time–frequency analysis method commonly used in signal processing and fault diagnosis [28]. The MEMD technique is used for multichannel signal fusion and detects automatic transmission system faults based on vibration monitoring [29]. Currently, extreme learning machines (ELM), support vector machines (SVM), and general regression neural networks (GRNN) are common machine learning classification methods. The SVM was used in the fault diagnosis of high-voltage circuit devices, and the fault diagnosis model exhibited good accuracy and efficiency [30]. An ELM was used in a three-group rolling bearing fault diagnosis experiment, and could accurately identify the location of the fault distribution [31].

The innovation of this study is the application of MEMD to hyperspectral data processing to explore the formation of the intrinsic mode function (IMF) with spectral information. More characteristic bands were mined from several IMFs and combined with machine learning classification algorithms, ELM, SVM, and GRNN, to establish models to identify the types of Cu and Pb pollution elements.

As one of the main crops in China and worldwide, maize has been affected by heavy metal pollution in some areas [32]. During the growth cycle of corn, leaves are highly sensitive to heavy metal stress [33]. The higher the residual amounts of heavy metals in the leaves, the higher the content of heavy metals in the fruit. Leaves are the main organs involved in plant photosynthesis. Therefore, it is necessary to detect and analyze heavy metal content in maize leaves under different concentrations of Cu and Pb stress during the plant growth stage [34].

Therefore, in this study, starting at the leaf scale and taking copper and lead pollution elements as examples, the objectives of this study were to (1) acquire hyperspectral data of corn leaves; (2) expand and highlight the spectral characteristics of spectrum processed using MEMD; (3) fully exploit the spectral information of the vegetation with the successive projections algorithm (SPA), competitive adaptive reweighted sampling (CARS), and iteratively retaining informative variables (IRIV) used to screen sensitive bands; (4) establish an effective hyperspectral model for distinguishing copper and lead elements by combining the machine learning classification algorithms: SVM, ELM, and GRNN.

This study provides a new idea and method for further exploring the monitoring and identification of heavy metal contaminated elements in large-scale environmental soils under vegetation coverage.

## 2. Materials and Methods

### 2.1. Experimental Design and Data Acquisition

#### 2.1.1. Experimental Design

The experiment used pot cultivation to raise corn plants and was conducted at the China University of Mining and Technology-Beijing. To avoid the effect of varietal changes on the spectrum, all corn seeds were selected from “Minuo-8”. The selected seeds were close in size and full of particles, and they were placed in a 150A digital display biochemical incubator for germination treatment in advance. Analytical grade CuSO_4_·5H_2_O and Pb(NO_3_)_2_ with higher purity and fewer interfering impurities were used as heavy metal Cu^2+^ and Pb^2+^ coercion reagents, respectively. During the growth of corn plants, the same concentrations of NH_4_NO_3_, KH_2_PO_4_, and KNO_3_ nutrient solutions were added to make the concentrations of N, P, and K in the soil 100, 30, and 150 mg/kg, respectively. In the process of corn cultivation, to maintain sufficient soil moisture, watering occurred once a day, 200 mL each time, with uninterrupted ventilation throughout the day, which was conducive to the growth environment. In addition, the contents of N, P, and K; pH value, soil moisture; soil particle size were the same in the experiment. Maize plants were maintained in a normal environment to avoid external factors affecting the experiment. According to GB15618-2018: Soil Environmental Quality Soil Contamination Risk Control Standards for Agricultural Land (Trial) [35], seven copper and lead stress gradients were randomly set in the cultivation experiment, and the stress concentrations were set at 50, 100, 150, 400, 600, 800, and 1000 μg/g, labeled as Cu(50), Cu(100), Cu(150), Cu(400), Cu(600), Cu(800), Cu(1000), Pb(50), Pb(100), Pb(150), Pb(400), Pb(600), Pb(800), and Pb(1000). Three pots of corn were planted for each stress concentration for a total of forty-two pots.

#### 2.1.2. Spectral Data Acquisition

The spectral data of corn leaves were measured indoors using an SVC HR-1024i spectrometer produced by Spectrum Vista Corporation in the United States. In the process of spectrum collection, the indoor light source was provided by a 50 W halogen lamp with an illumination angle of 45° and fixed on a bracket. The field of view of the fiber optic probe was set to 25°, the probe was perpendicular to the blade surface, and the vertical distance was less than 5 cm. To prevent the influence of other background factors on the corn leaf spectra, the corn leaves were cut and measured on an experimental bench covered with a black cloth. The spectrum of corn leaves was collected from 10:00–14:00 on 19 July 2017. Because of the long collection time of the leaf spectrum, to eliminate the noise generated by the spectrometer during the collection process, a standard whiteboard was used to calibrate the spectrum before and after the collection of each maize leaf spectrum to ensure the stability of the measurement. A standard Teflon whiteboard was used as the background for whiteboard calibration. Because old leaves grow for a long time and the accumulation of heavy metals is more serious, old leaves were selected for this study. Therefore, during spectrum collection, the spectrum of old maize leaves under each stress concentration in each parallel group was measured 3 times, and 9 sets of data were obtained for each stress concentration, totaling 135 sets of spectral data.

#### 2.1.3. Chemical Detection of Heavy Metal Content

Chemical analysis of the Cu and Pb content in the leaves was performed immediately after leaf spectral data acquisition. In this study, a wet chemical method was used to determine heavy metal content. The specific steps were as follows: (1) The corn leaves were washed with distilled water three times and dried on clean absorbent paper. After the moisture was absorbed, the leaves were dried in an oven at 80 °C for 24 h. (2) The leaves were removed and cooled to a constant weight for later use. The dried corn leaves were crushed using a high-speed pulverizer, produced by Jinhua, Zhejiang, China, and the larger blade fragments were removed using a 120-mesh nylon sieve. (3) One gram of the sieved sample particles was weighed and placed in a 150 mL high beaker, 20 mL of nitric acid was added, and then it was covered with a watch glass and left overnight. Twenty microliters of nitric acid was added to the high beaker, and then it was placed on an electric furnace for heating at a low temperature until the sample dissolved and then cooled down slightly. Then, 5 mL perchloric acid was added, the heating temperature gradually increased, and then 5 mL nitric acid was added and then heated until the solution changed from reddish-brown to colorless. The temperature was increased, and heating was continued until a large amount of thick white smoke emerged. The white smoke was driven out, and light yellow flocs appeared, after which the heating was stopped, and the solution cooled to room temperature. Two microliters of nitric acid solution was added to warm slightly, and then cooled to room temperature. The sample digestion solution was transferred to a 25 mL volumetric flask to a constant volume and shaken well. Then, the content of copper ions and lead ions was measured with an inductively coupled plasma optical emission spectrometer (ICP-OES). The analysis conditions of the ICP-OES: emission power was 1200 W; the cooling gas flow was 12 L/min; the auxiliary gas flow was 0.5 L/min; the normal injection time was 15 s; the number of measurements was 3 times.

### 2.2. Theory and Method

#### 2.2.1. Multivariate Empirical Mode Decomposition (MEMD)

Mode decomposition is a signal decomposition method that can obtain more stable time series signals. Empirical mode decomposition (EMD) was proposed by Huang et al. [36], in 1998, for nonstationary signal processing. The disadvantage of EMD is that for multi-input signals, each channel signal must be solved separately. Thus, the number and frequency scale of the intrinsic mode function (IMF) decomposed by different channel signals are different. To address this shortcoming, Rehman et al. [37] proposed a multivariate empirical mode decomposition (MEMD) suitable for multichannel signal data analysis by improving the EMD. The MEMD can decompose multiple signals into the same number of IMFs. This theory effectively guarantees simultaneous joint analysis of multi-input channel signals. The MEMD solves the problems of modal mixing and scale misalignment of the layers in the EMD transformation and is adaptive.

In this study, MEMD was applied to hyperspectral data processing, and the obtained spectral data of corn leaves were processed by MEMD transformation to explore the formation of MEMD processing technology with spectral information. The formula for the MEMD transformation is briefly described here. The decomposition and construction processes of MEMD have been described in detail in the literature [37]. The steps for the MEMD calculation are as follows:

(1) Let the n-dimensional input sequence vector be $v{\left(t\right)}_{i=1}^{T}=\left\{v_{1}\left(t\right),v_{2}\left(t\right),\ldots ,v_{n}\left(t\right)\right\}$, where *v_n_*(*t*) represents the input time series vector; *T* is the length of the input sequence. The angle obtained by sampling the input sequence is $\theta^{k}=\left\{\theta_{1}^{k},\theta_{2}^{k},\ldots ,\theta_{n-1}^{k}\right\}$, where $\theta_{n-1}^{k}$ represents the angle of the sampling sequence, and the set of direction vectors is $x^{\theta_{k}}=\left\{x_{1}^{k},x_{2}^{k},\ldots ,x_{n-1}^{k}\right\}$. *K* indicates that there are *K* directional vectors on the sphere; $x_{n-1}^{k}$ represents the direction vector;

(2) The Hammersley sequence sampling method is used to uniformly sample the (n − 1)-dimensional hypersphere to obtain *K* sets of direction vectors;

(3) Compute the set ${\left\{p^{\theta_{k}}\left(t\right)\right\}}_{k=1}^{K}$ of projections of multiple input sequences ${\left\{v\left(t\right)\right\}}_{i=1}^{T}$ on the *K*-group direction vector $x^{\theta_{k}}$;

(4) Calculate the instantaneous moments $t_{h}^{\theta_{k}}$ corresponding to all extreme value points in the projection set ${\left\{p^{\theta_{k}}\left(t\right)\right\}}_{k=1}^{K}$;

(5) Interpolation calculation $t_{h}^{\theta_{k}},v\left(t_{h}^{\theta_{k}}\right)$ to obtain the multivariate envelope curve $e^{\theta_{k}}{\left(t\right)}_{k=1}^{K}$;

(6) The mean value *m*(*t*) of the entire set of direction vector envelope curves is calculated:

(1) $$m\left(t\right)=\frac{1}{k}\sum_{k=1}^{K}e^{\theta_{t}}\left(t\right)$$

(7) *d*(*t*) is obtained according to the equation *d*(*t*) = *v*(*t*) − *m*(*t*). If *d*(*t*) satisfies the iteration termination condition, it is considered to be the current IMF component; otherwise, it goes to step three to continue the decomposition.

The stopping criteria for multiple IMFs are as follows: (i) the difference between the sum of the maximum and minimum points in the IMF sequence and the number of zero-crossing points is 0 or 1; (ii) the mean value of the upper and lower envelopes of the IMF sequence is zero at any time.

The MEMD is used to decompose the input spectral signal *v*(*t*) to obtain several different IMFs. As shown in Equation (2):

(2) $$v\left(t\right)=\overset{M}{\underset{m-1}{\sum }}c_{m}\left(t\right)+r\left(t\right)$$

In Formula (2), $c_{m}\left(t\right)$ represents the *m*th IMF, and *r*(*t*) represents the decomposition residual.

Compared to the traditional EMD algorithm, the MEMD algorithm is more robust. Several IMFs obtained using the MEMD transform can be used to effectively analyze the characteristics of the input signal.

#### 2.2.2. Successive Projections Algorithm (SPA)

The successive projection algorithm (SPA) is a forward-cycling method for wavelength selection [38]. The principle of the algorithm is that in the first iteration, starting from any wavelength, each cycle calculates the projection of that wavelength on all unselected wavelengths, compares the size, selects the largest projection vector corresponding to the variable to be selected, and finally iterates until the best number of wavelength variables is selected [39]. This method has been increasingly used in screening and extracting sensitive variables and can effectively eliminate the problem of spectral information covariance.

#### 2.2.3. Iteratively Retaining Informative Variables (IRIV)

Iteratively retaining informative variables (IRIV) is a new variable selection algorithm proposed by Yun et al. [40]. It uses a new sampling algorithm to obtain a series of random combinations of variables, conducts a modeling analysis on each of these combinations, and observes the changes in the prediction error of interactive verification when a variable exists and does not exist in the model. Based on this change, and according to the idea of model population analysis (MPA), the variables are divided into four categories: interference-informative variables (IIV), noninformative variables (NIV), weakly informative variables (WIV), and strong-informative variables (SIV). Each variable is analyzed in turn, and the final selected variables are all WIVs and SIVs to obtain the optimal characteristic wavelength variable [41].

#### 2.2.4. Competitive Adaptive Reweighted Sampling (CARS)

Competitive adaptive reweighted sampling (CARS) is a method for variable selection and elimination that was established based on the survival of the fittest law of organisms in nature [42]. CARS selects the wavelength points with a large absolute value of the regression coefficient in the partial least squares (PLS) model through adaptive reweighted sampling (ARS) technology, removes the wavelength points with small weights, and selects N by cross-validation. The subset with the smallest RMSECV in the PLS subset model and the variables contained in this subset are the optimal variable combinations. Using this method, the optimal band combination can be selected effectively [43].

#### 2.2.5. Extreme Learning Machines (ELM)

The extreme learning machine (ELM) algorithm was proposed by Huang et al. [44]. As a new, fast, and efficient machine learning algorithm, the algorithm is a supervised classification algorithm based on a single hidden layer feedforward neural network. Compared with traditional machine learning algorithms, in the ELM algorithm, the weight parameters between the input and implicit layers and the bias parameters of the implicit layer need not be adjusted repeatedly by constant iterative computations. Therefore, the number of calculations of the algorithm is reduced, the training time is shortened, and the calculation efficiency is improved. Moreover, the algorithm has a better generalization performance and can better meet the dual requirements of accuracy and computational speed of the classification algorithm.

#### 2.2.6. Support Vector Machines (SVM)

As an efficient machine learning algorithm, the support vector machine (SVM) algorithm is often used in regression analysis, target classification, etc. [45]. The SVM focuses on machine learning laws in the case of limited samples. The basic idea is to transform a vector of n-dimensional inputs into a high-dimensional feature space. Then, the optimal hyperplane is found in the high-dimensional eigenspace such that the invisible test pattern prediction classification error is minimized, and the two types of data are separated as accurately as possible.

#### 2.2.7. General Regression Neural Network (GRNN)

The general regression neural network (GRNN) algorithm was proposed by American scholar Donald F. Specht in 1991 [46]. The algorithm is a local approximation network that is based on mathematical statistics. The algorithm is theoretically based on nonlinear regression analysis and is a radial neural network with a high degree of parallelism at the same time. In the training calculation, only a small number of weights and thresholds need to be modified. Therefore, the GRNN algorithm is simple, highly accurate, and has a strong nonlinear convergence and operational speed. The GRNN algorithm has been widely used for prediction, classification, and recognition.

#### 2.2.8. Accuracy Evaluation Method

The accuracy of the model constructed by the classification method used in this study was evaluated by accuracy (*A*), precision (*P*), recall (*R*), and a comprehensive evaluation *F*-score [47]. The calculation formulas are Equations (3)–(6):

(3) $$A=\frac{N_{TP}+N_{TN}}{N_{TP}+N_{TN}+N_{FP}+N_{FN}}$$

(4) $$P=\frac{N_{TP}}{N_{TP}+N_{FP}}$$

(5) $$R=\frac{N_{TP}}{N_{TP}+N_{FN}}$$

(6) $$F=\frac{2P\times R}{P+R}$$

where *N_TP_* represents the number of correctly classified positive examples; *N_FP_* represents the number of misclassified positive examples; *N_TN_* represents the number of correctly classified negative examples; *N_FN_* represents the number of misclassified negative examples.

#### 2.2.9. Workflow

**Figure 1:**
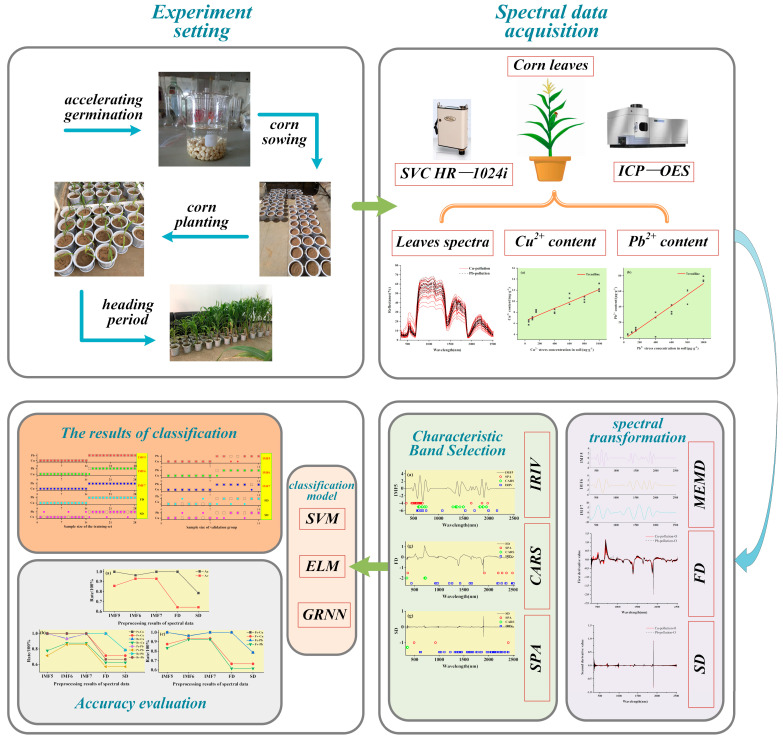
Flowchart of the study.

The workflow of this study is illustrated in Figure 1. (1) Maize was planted in pots, simulating maize growth experiments under heavy metal Cu and Pb stress, and maize leaves were collected at the heading stage. (2) The spectral data of the maize leaves were measured using an SVC HR-1024i spectrometer (Spectra Vista Corporation, Poughkeepsie, NY, USA). The Cu and Pb ion contents in the leaves were determined using an inductively coupled plasma optical emission spectrometer (ICP-OES, PerkinElmer, Waltham, MA, USA). (3) The MEMD algorithm was introduced to transform the spectral data, while the first derivative (FD) and second derivative (SD) transforms were used to preprocess the spectral data. (4) Characteristic bands were selected using the SPA, CARS, and IRIV algorithms. (5) Three machine learning algorithms, SVM, ELM, and GRNN, were used to distinguish the categories of Cu and Pb. The best discriminative model was evaluated using *A*, *P*, *R*, and *F*-score.

## 3. Results and Discussion

### 3.1. Data Preprocessing and Analysis

#### 3.1.1. Heavy Metal Content of Corn Leaves

**Figure 2:**
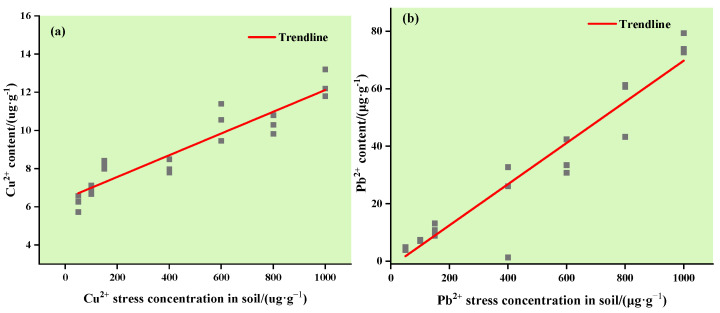
Relationship between the Cu^2+^ and Pb^2+^ stress gradient in the soil and its content in corn leaves: (**a**) changes in the Cu^2+^ content in maize leaves under Cu stress; (**b**) changes in the Pb^2+^ content in maize leaves under Pb stress.

The Cu^2+^ and Pb^2+^ content in corn leaves under different concentrations of Cu and Pb stress are shown in Figure 2. As shown in Figure 2a, with an increase in the copper stress concentration, the overall trend of Cu^2+^ content in maize leaves gradually increased. As shown in Figure 2b, the Pb^2+^ content in maize leaves increased faster with increasing lead stress concentration. This shows that the absorption and enrichment of Pb^2+^ in the leaves were significant.

#### 3.1.2. Spectral Data Preprocessing and Analysis

**Figure 3:**
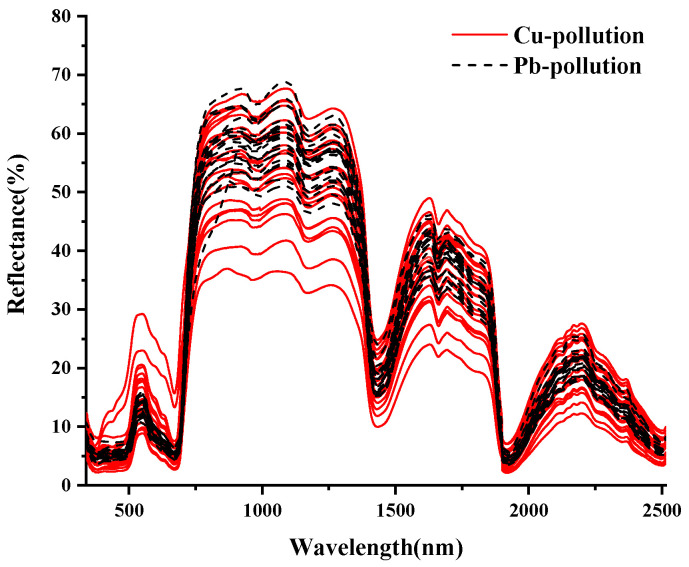
The average spectral curve of corn leaves under different concentrations of Cu^2+^ and Pb^2+^. **Notes:** Cu pollution represents the spectrum of leaves under copper stress, and Pb pollution represents the spectrum of leaves under lead stress.

The final spectrum data were obtained by removing outliers and averaging, as shown in Figure 3. The spectral features of vegetation in the visible (VIS: 350–700 nm) portion of the spectrum are typically driven by leaf pigments [48]. The increased reflectance of leaves in the near and mid-infrared regions is because leaves absorb less light [49]. In conclusion, the overall spectral curves of maize leaves under Cu and Pb stress showed basically the same overall trend, with high similarity and no significant differences [50]. Therefore, the types of Cu and Pb pollution elements could not be accurately identified by calculating the average value of the spectral curve. It is well known that spectral transformations play an important role in hyperspectral modeling, and the preprocessing of spectral data can effectively improve the effectiveness of classification.

**Figure 4:**
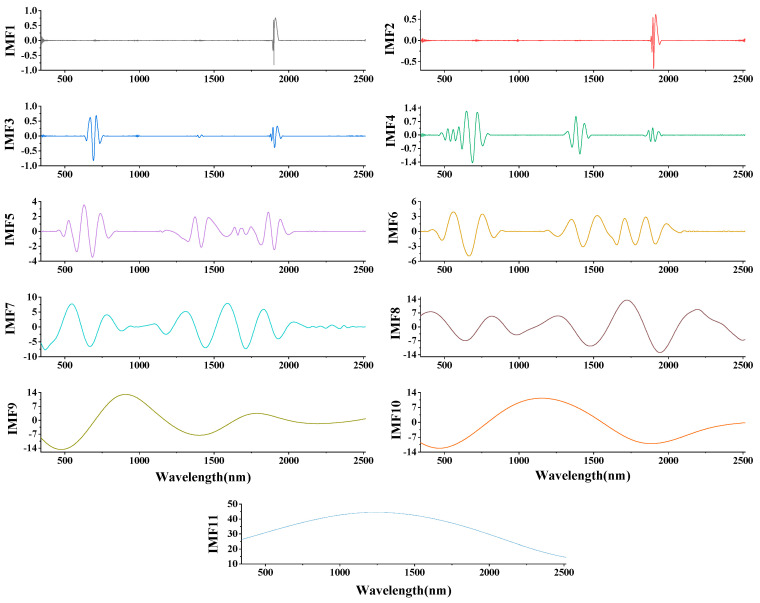
Results of MEMD processing of old leaves of Cu(100) − 1.

As is known, spectral transformation plays an important role in hyperspectral modeling, and preprocessing spectral data can effectively improve the classification effect [51]. In this study, multivariate empirical mode decomposition (MEMD), first derivative (FD), and second derivative (SD) technologies were used to preprocess spectral data. The spectral data were decomposed by MEMD into ten IMF components and one trend component (*r*). Each component contained rich spectral information, which was convenient for fully mining the hidden spectral curve information. As shown in Figure 4, taking the MEMD transformation of the leaf spectrum of Cu(100)-1 as an example, the results of the MEMD transformation are shown. As the scale of MEMD increased, the spectral response gradually decreased. Among them, IMF1-IMF4 were noisy and did not exhibit good mode separation. The IMF8-IMF10 components tended to be smooth, the fluctuations were small, the useful information contained gradually decreased, and the last component *r* was the trend component. The IMF5-IMF7 components were well decomposed, smooth, and prominent, further highlighting some inconspicuous characteristic peaks in the spectral curve. It is difficult to obtain useful spectral information at both low and high decomposition scales. Therefore, in this study, the IMF5-IMF7 components were selected for further research.

**Figure 5:**
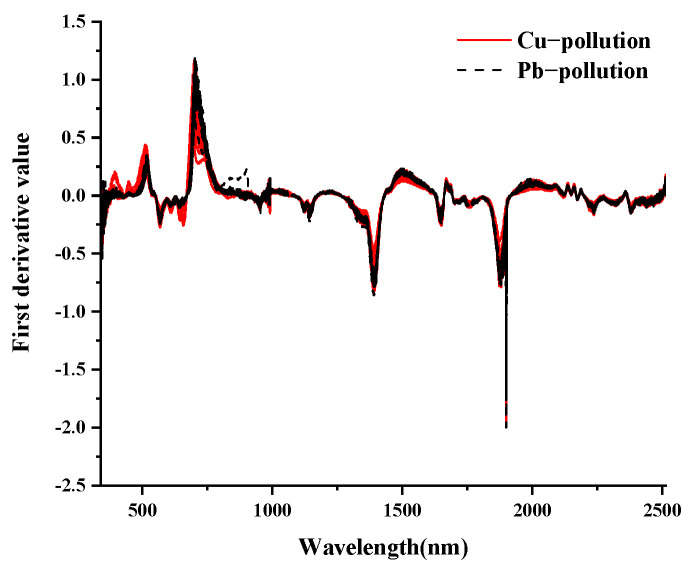
First−order differential transformation of spectrum.

**Figure 6:**
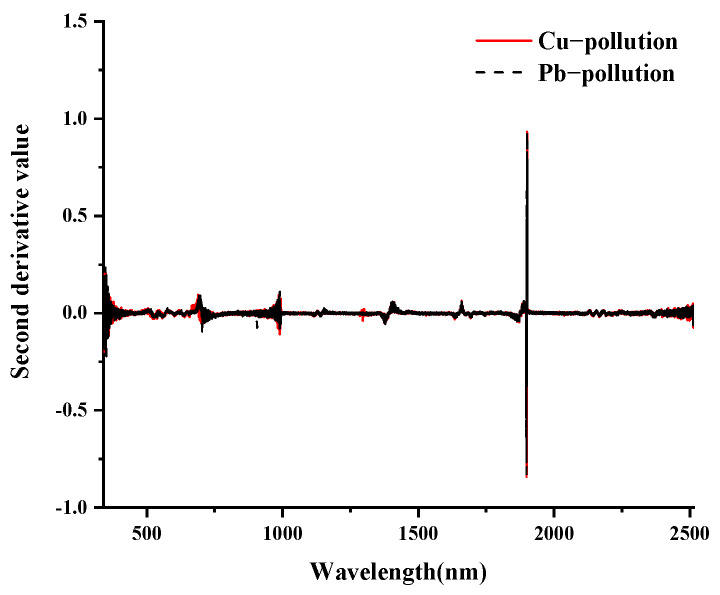
Second−order differential transformation of spectrum.

The FD and SD transformations of the spectrum can solve the overlapping peaks of the spectrum curve, enhance the difference between the spectra, and effectively enhance and highlight subtle changes in the spectrum. The results of the FD and SD transformations of the spectra are shown in Figure 5 and Figure 6. For the meaning of the legend in the figure, refer to the notes in Figure 3.

The results of this study demonstrate that the MEMD transform can effectively highlight characteristic spectra. A possible reason for this is that MEMD, as a time-scale decomposition algorithm of signals, can decompose the spectrum in the time domain, and related studies have also proved similar conclusions [52]. Relevant research also proves that signal decomposition in the frequency and time domains can effectively highlight spectral information and obtain spectral characteristics [53,54].

### 3.2. Characteristic Band Acquisition Using SPA, CARS, and IRIV

**Figure 7:**
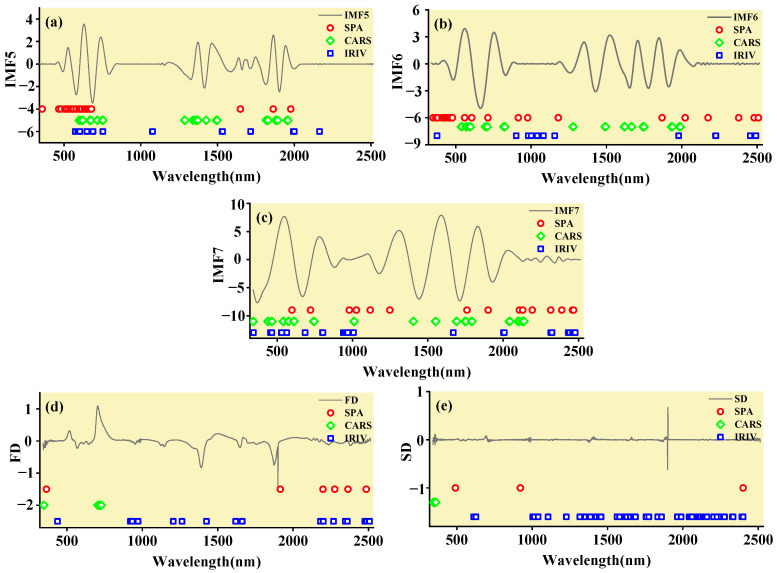
The position of the characteristic wavelengths of leaf spectrum. (**a**) Perform MEMD transformation on the spectrum to obtain the IMF5 component, and filter the characteristic bands; (**b**) Perform MEMD transformation on the spectrum to obtain the IMF6 component, and filter the characteristic bands; (**c**) Perform MEMD transformation on the spectrum to obtain the IMF7 component, and filter the characteristic bands; (**d**) Perform FD transformation on the spectrum to filter characteristic bands. (**e**) Perform SD transformation on the spectrum to filter characteristic bands.

Determining the best characteristic band to monitor plant parameters from a large amount of hyperspectral band data is the most critical step in spectroscopy [55]. To effectively reduce the dimensions of the spectral data and improve the performance of the classification model, the successive projections algorithm (SPA), competitive adaptive reweighted sampling (CARS), and iteratively retaining informative variables (IRIV) were used to screen the optimal characteristic bands [56]. The characteristic wavelength bands were selected for the components IMF5, IMF6, and IMF7 obtained after MEMD transformation and the spectrum after FD and SD transformation, and the obtained characteristic wavelength distribution is shown in Figure 7. It can be seen from Figure 7a–c that for the IMF components obtained by MEMD transformation, most of the characteristic bands selected by SPA, CARS, and IRIV were concentrated in the VIS (380–780 nm) region. The 670–760 nm region, where the reflectance changes rapidly, is called the “red edge” of vegetation [57].

It can be seen from Figure 7d,e that after the FD and SD transformations of the spectrum, only a small number of bands could be screened by SPA and CARS, whereas a large number of characteristic bands may be extracted by IRIV. In the following classification model, we used the spectral value of each feature band as the input data for the model.

### 3.3. Identification of Copper and Lead Elements

In this study, all leaf samples (*n* = 42) collected were randomly divided into three datasets according to the stress gradient, and the number of samples in each dataset was the same. Two sets of data were randomly selected as the *calibration* group (*n* = 28), and the remaining data (*n* = 14) were used to verify the accuracy of the classification model. After screening the optimal characteristic bands by SPA, CARS, and IRIV combined with the ELM, SVM, and GRNN classification methods to construct a differentiation model, it was used to distinguish the categories of heavy metals Cu and Pb. The final discrimination results were evaluated using accuracy (*A*), precision (*P*), recall (*R*), and *F*-score.

#### 3.3.1. SVM Classification and Discrimination Model Based on the Optimal Wavelength

**Figure 8:**
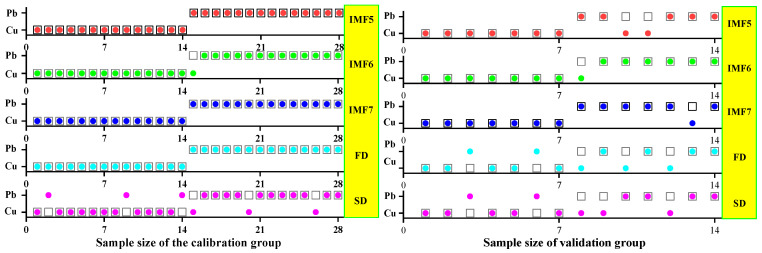
Discrimination results of Cu and Pb by SPA-SVM.

**Figure 9:**
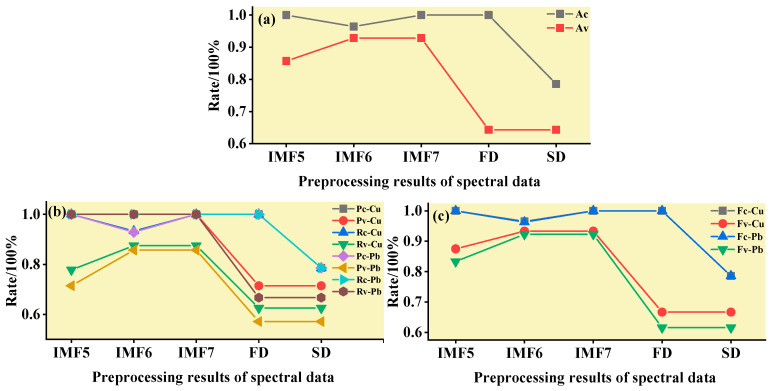
Evaluation of the discrimination accuracy of Cu and Pb by SPA-SVM. (**a**) The accuracy of the model; (**b**) The precision and recall of the model; (**c**) Comprehensive evaluation *F*-score of the model.

**Figure 10:**
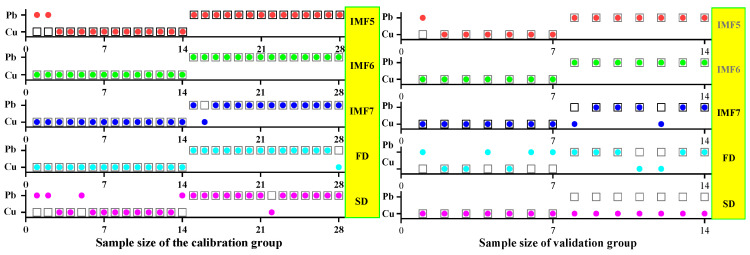
Discrimination results of Cu and Pb by CARS-SVM.

**Figure 11:**
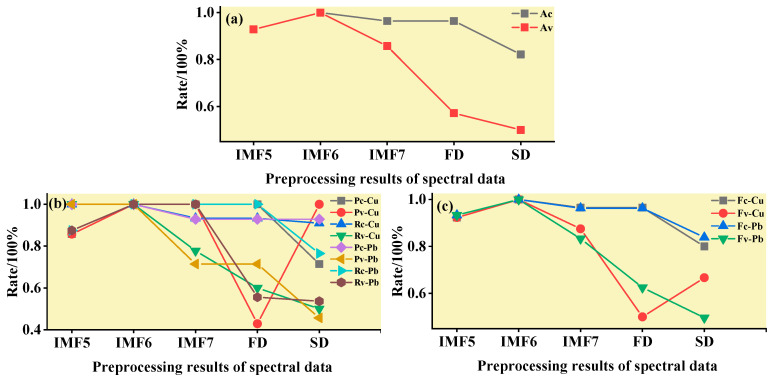
Evaluation of the discrimination accuracy of Cu and Pb by CARS-SVM. (**a**) The accuracy of the model; (**b**) The precision and recall of the model; (**c**) Comprehensive evaluation *F*-score of the model.

**Figure 12:**
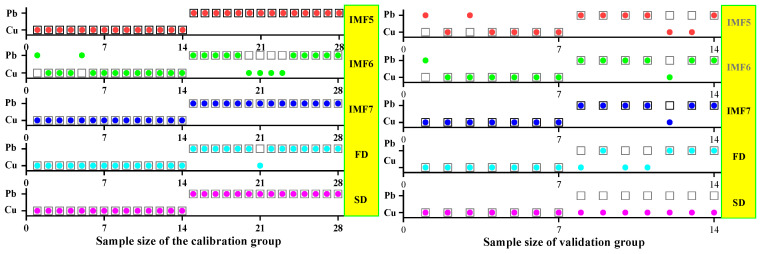
Discrimination results of Cu and Pb by IRIV-SVM.

**Figure 13:**
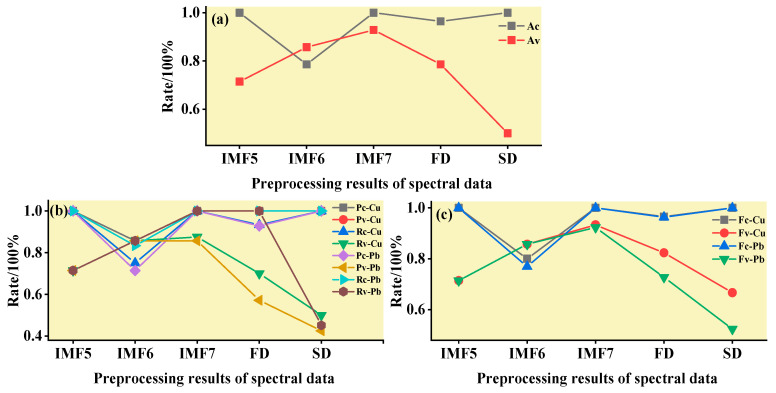
Evaluation of the discrimination accuracy of Cu and Pb by IRIV-SVM. (**a**) The accuracy of the model; (**b**) The precision and recall of the model; (**c**) Comprehensive evaluation *F*-score of the model.

The practical role of the characteristic bands selected by the three band screening methods was evaluated in this step. Based on the above characteristic wavelengths, the MEMD-SPA-SVM, MEMD-CARS-SVM, and MEMD-IRIV-SVM models were established to distinguish between Cu and Pb types. Meanwhile, the common spectral preprocessing methods, FD and SD, were used instead of MEMD in the three previous models to construct the same models for comparative analysis. The results are presented in Figure 8, Figure 9, Figure 10, Figure 11, Figure 12 and Figure 13. The abscissa represents the sample size, the ordinate represents the type of copper and lead elements, the squares represent the correct element category, and the circles represent the classification results of the copper and lead elements calculated by the model. When a circle falls into a square, this means that the classification was correct; otherwise, it means that the classification was wrong. The yellow band indicates the corresponding spectral transformation. Line charts show the accuracy evaluations of the various models.

(1) MEMD-SPA-SVM

The spectral data were preprocessed by MEMD transformation, the characteristic bands were screened by SPA, and the model established by combining with SVM had a better ability to distinguish between the Cu and Pb categories (Figure 8). The accuracy (*R*) and *F*-score of the modeling and validation sets were both greater than 0.8, and the precision (*P*) and recall (*R*) of the Cu and Pb categories were approximately 0.8 (Figure 9). Compared with the model established by FD and SD transformation, when distinguishing between Cu and Pb species, although the accuracy of the *calibration* group was higher, the accuracy of the validation group was less than 0.8, and the differentiation effect was poor.

(2) MEMD-CARS-SVM

The spectral data were preprocessed by MEMD transformation, the characteristic bands were screened by CARS, and the model established by combining with SVM had a better ability to distinguish between the Cu and Pb categories (Figure 10). For both the modeling set and the validation set, the copper and lead elements could fall into the exact category. The accuracy (*R*) and *F*-score of the modeling and validation sets were both greater than 0.8, and the precision (*P*) and recall (*R*) of the Cu and Pb categories were also approximately 0.8 (Figure 11). Compared with the model established by FD and SD transformation, when distinguishing between Cu and Pb species, although the accuracy of the *calibration* group was higher, the accuracy of the validation group was less than 0.8, and the differentiation effect was poor. Differential transformation of the spectrum could not distinguish the categories of copper and lead elements well.

(3) MEMD-IRIV-SVM

In contrast, in the model established by IRIV screening of the characteristic bands, only the IMF7 component had a better effect in distinguishing Cu and Pb species (Figure 12). The accuracy (*R*) and *F*-score of the modeling and validation sets were both greater than 0.8, and the precision (*P*) and recall (*R*) of the Cu and Pb categories were also approximately 0.8 (Figure 13). Compared with the model established by FD and SD transformation, although the accuracy of the *calibration* group was higher, the accuracy of the validation group was also less than 0.8, and the differentiation effect was poor.

This may mean that the feature bands extracted after FD and SD transformations were suitable for modeling samples but not for verification samples, and they could not accurately distinguish the categories of Cu and Pb. Therefore, the MEMD-SPA-SVM and MEMD-CARS-SVM models had better accuracy and advantages in distinguishing Cu and Pb species. Different spectral data preprocessing methods result in different classification accuracies. Compared to the commonly used FD and SD spectral preprocessing methods, the model established from the data processed by the MEMD transform provided better classification and recognition results. This improvement was due to the advantages of MEMD. The sensitive bands extracted after MEMD transformation of the spectrum not only revealed the correlation between the leaf spectrum and the intrinsic mode function (IMF) but also revealed subtle changes in the leaf spectrum under heavy metal stress. The MEMD transformation of the spectrum was combined with SPA, CARS, and IRIV to improve the performance of the SVM method in distinguishing Cu and Pb species. This may be because the IMF5-7 components selected by the MEMD transform removed interference such as baseline shift and artificial noise.

#### 3.3.2. ELM Classification and Discrimination Model Based on the Optimal Wavelength

**Figure 14:**
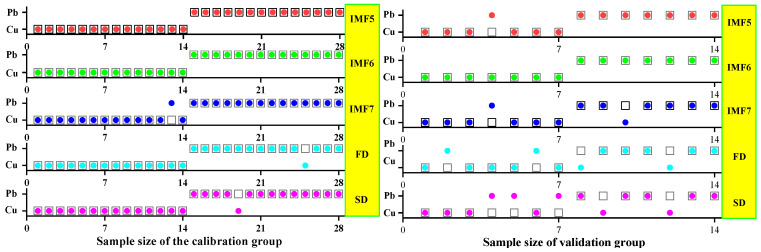
Discrimination results of Cu and Pb by SPA-ELM.

**Figure 15:**
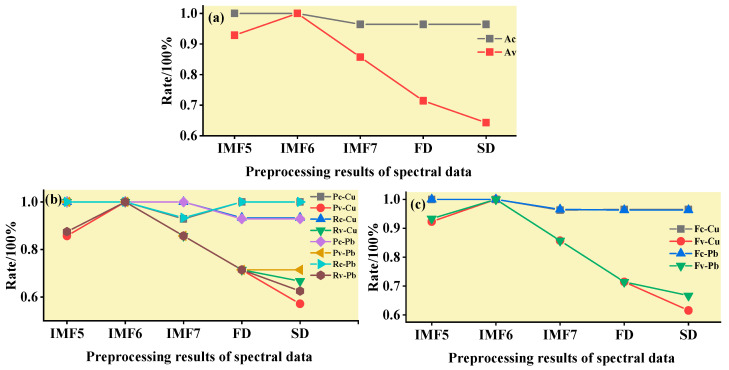
Evaluation of the discrimination accuracy of Cu and Pb by SPA-ELM. (**a**) The accuracy of the model; (**b**) The precision and recall of the model; (**c**) Comprehensive evaluation *F*-score of the model.

**Figure 16:**
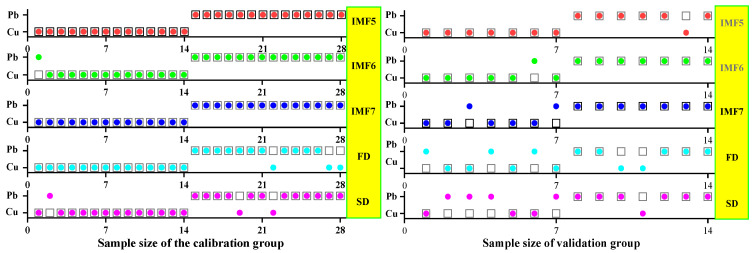
Discrimination results of Cu and Pb by CARS-ELM.

**Figure 17:**
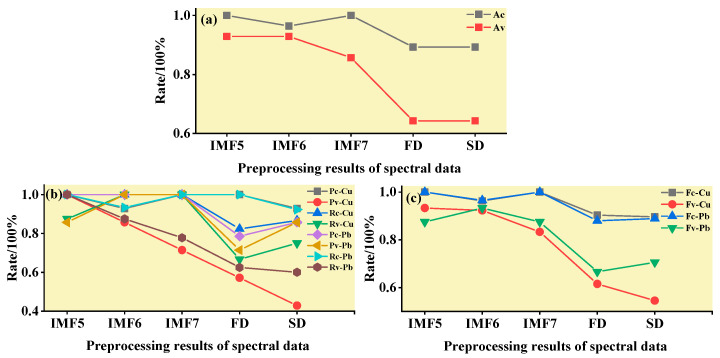
Evaluation of the discrimination accuracy of Cu and Pb by CARS-ELM. (**a**) The accuracy of the model; (**b**) The precision and recall of the model; (**c**) Comprehensive evaluation *F*-score of the model.

**Figure 18:**
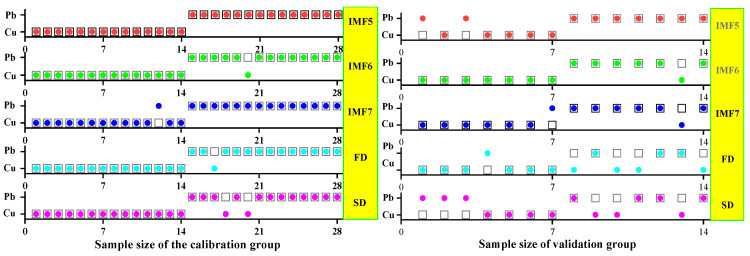
Discrimination results of Cu and Pb by IRIV-ELM.

**Figure 19:**
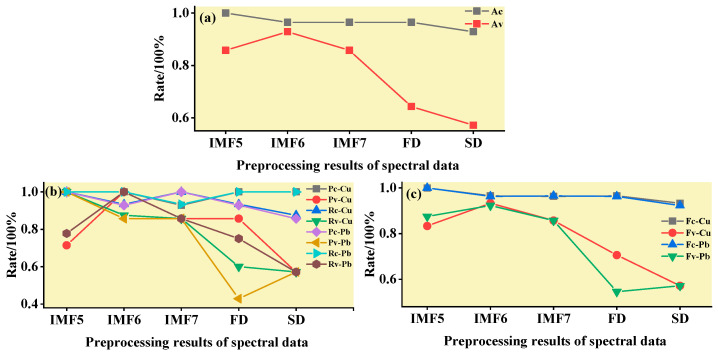
Evaluation of the discrimination accuracy of Cu and Pb by IRIV-ELM. (**a**) The accuracy of the model; (**b**) The precision and recall of the model; (**c**) Comprehensive evaluation *F*-score of the model.

In this step, three models were established based on the aforementioned characteristic wavelengths to distinguish Cu and Pb types: the MEMD-SPA-ELM, MEMD-CARS-ELM, and MEMD-IRIV-ELM models. The FD and SD methods were used instead of the MEMD in the three previous models to construct the same models for comparative analysis. The results of this distinction are shown in Figure 14, Figure 15, Figure 16, Figure 17, Figure 18 and Figure 19, and the coordinates in the figure are shown in Section 3.3.1.

(1) MEMD-SPA-ELM

After the MEMD transformation of the spectrum, SPA was used to screen the characteristic bands, and the model established by combining with ELM could distinguish Cu and Pb elements (Figure 14). The accuracy (*A*) and *F*-score of its *calibration* group and validation group were both greater than 0.8 (Figure 15). The classification precision (*P*) and recall (*R*) of Cu and Pb were also above 0.8, which is considered a good result. It is worth noting that for the model established by FD and SD transformations, when distinguishing Cu and Pb species, although the calibration group accuracy was higher, the validation group accuracy was less than 0.8; therefore, it was not successful enough to distinguish Cu and Pb species.

(2) MEMD-CARS-ELM

After the MEMD transformation of the spectrum, CARS was used to screen the characteristic bands, and the model established by combining with ELM could also distinguish Cu and Pb elements well (Figure 16). Almost all copper and lead elements were accurately classified. The accuracy (*A*) and *F*-score of its calibration group and validation group were both greater than 0.8 (Figure 17). The classification precision (*P*) and recall (*R*) of Cu and Pb were also above 0.8, which is considered a good result. It is worth noting that for the model established by FD and SD transformation, when distinguishing Cu and Pb species, although the calibration group accuracy was higher, the validation group accuracy was less than 0.8; therefore, it was not successful enough to distinguish Cu and Pb species.

(3) MEMD-IRIV-ELM

After the MEMD transformation of the spectrum, IRIV were used to screen the characteristic bands, and the model established by combining with ELM could distinguish Cu and Pb elements (Figure 18). The accuracy (*A*) and *F*-score of its *calibration* group and validation group were both greater than 0.8 (Figure 19). The classification precision (*P*) and recall (*R*) of Cu and Pb were also above 0.8, which is considered a good result. It is worth noting that for the model established by FD and SD transformation, when distinguishing Cu and Pb species, although the *calibration* group accuracy was higher, the validation group accuracy was less than 0.8; therefore, it was not successful enough to distinguish Cu and Pb species.

Therefore, the MEMD-SPA-ELM, MEMD-CARS-ELM, and MEMD-IRIV-ELM models had better advantages in distinguishing Cu and Pb species. This also shows that the components obtained after spectral preprocessing with MEMD can screen out bands that are sensitive to heavy metals, and they had the best overall performance in the differentiation of Cu and Pb species. For FD and SD transforms, it was not as good, and some could filter out only a few characteristic bands (Figure 7d,e). The lower performance of the resulting model may have been caused by differences in the characteristic band data.

#### 3.3.3. GRNN Classification and Discrimination Model Based on the Optimal Wavelength

**Figure 20:**
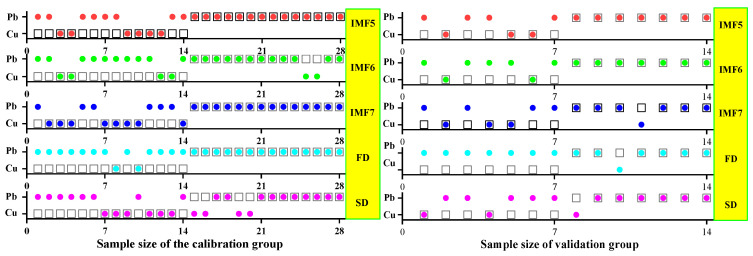
Discrimination results of Cu and Pb by SPA-GRNN.

**Figure 21:**
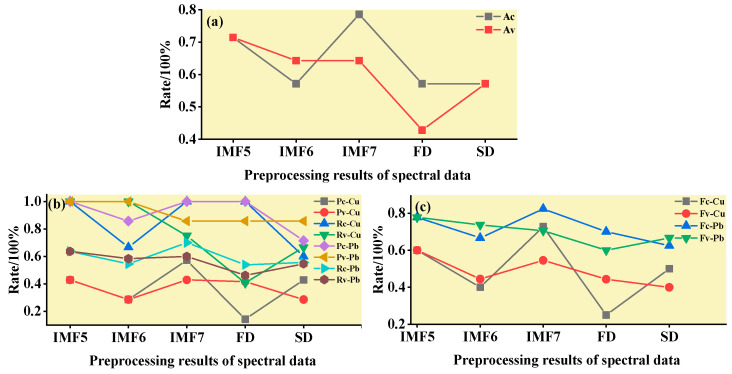
Evaluation of the discrimination accuracy of Cu and Pb by SPA-GRNN. (**a**) The accuracy of the model; (**b**) The precision and recall of the model; (**c**) Comprehensive evaluation *F*-score of the model.

**Figure 22:**
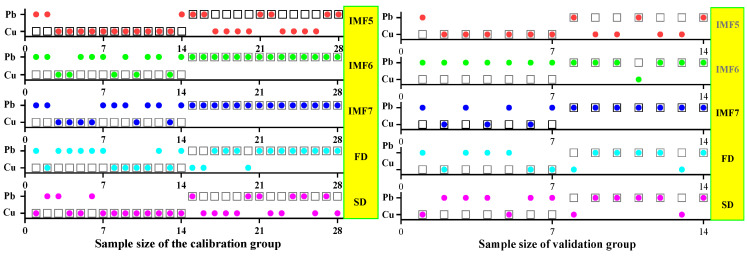
Discrimination results of Cu and Pb by CARS-GRNN.

**Figure 23:**
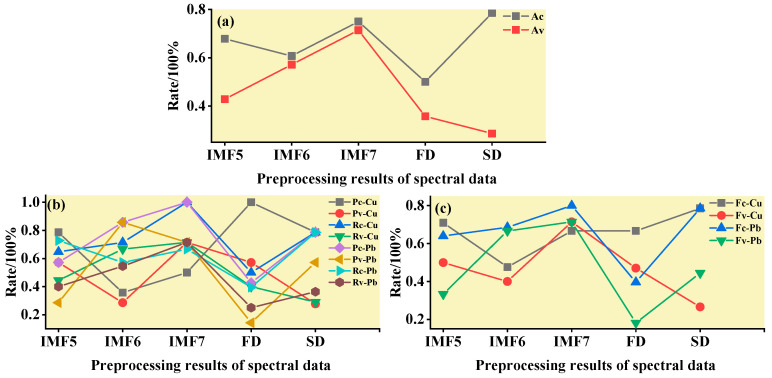
Evaluation of the discrimination accuracy of Cu and Pb by CARS-GRNN. (**a**) The accuracy of the model; (**b**) The precision and recall of the model; (**c**) Comprehensive evaluation *F*-score of the model.

**Figure 24:**
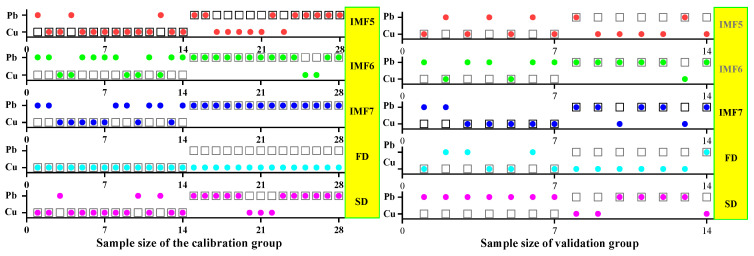
Discrimination results of Cu and Pb by IRIV-GRNN.

**Figure 25:**
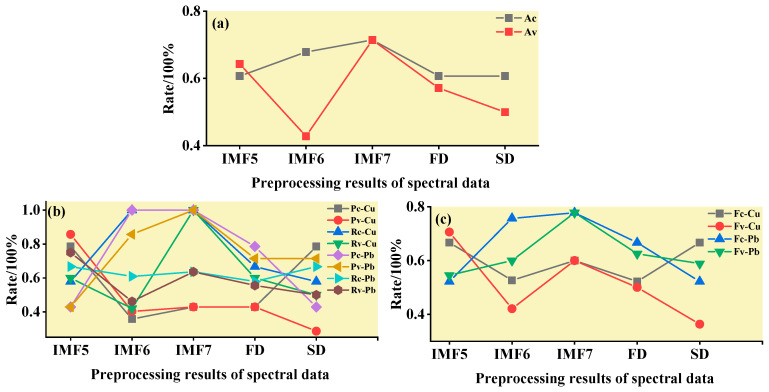
Evaluation of the discrimination accuracy of Cu and Pb by IRIV-GRNN. (**a**) The accuracy of the model; (**b**) The precision and recall of the model; (**c**) Comprehensive evaluation *F*-score of the model.

In this step, MEMD-SPA-GRNN, MEMD-CARS-GRNN, and MEMD-IRIV-GRNN models were established based on the above characteristic wavelengths to distinguish Cu and Pb types. Figure 20, Figure 21, Figure 22, Figure 23, Figure 24 and Figure 25 show the results of two-dimensional visual distinction and recognition of the Cu and Pb categories. The meanings of the symbols in the figures are presented in Section 3.3.1.

(1) MEMD-SPA-GRNN

After MEMD transformation of the spectrum, SPA was used to screen the characteristic bands, and the model that was established with GRNN could not distinguish Cu and Pb categories well (Figure 20). The accuracy (*A*) and *F*-score were low, both less than 0.8 (Figure 21). Moreover, many of the precision and recall rates for the identification of Cu and Pb species in the modeling and *calibration* group were lower than 0.6 (Figure 21), which we did not want to observe. It can be seen that the use of the GRNN algorithm to distinguish Cu and Pb categories was unsuccessful. However, compared with the model established by FD and SD transformations, the Cu and Pb species could not be accurately distinguished.

(2) MEMD-CARS-GRNN

After MEMD transformation of the spectrum, CARS was used to screen the characteristic bands, and the model that was established with GRNN could not distinguish Cu and Pb categories well (Figure 22). The accuracy (*A*) and *F*-score were low, both less than 0.8 (Figure 23). Moreover, many of the precision and recall rates for the identification of Cu and Pb species in the modeling and *calibration* group were lower than 0.6 (Figure 23), which we did not want to observe. It can be seen that the use of the GRNN algorithm to distinguish Cu and Pb categories was unsuccessful. However, compared with the model established by FD and SD transformations, the Cu and Pb species could not be accurately distinguished.

(3) MEMD-IRIV-GRNN

After MEMD transformation of the spectrum, IRIV were used to screen the characteristic bands, and the model that was established with GRNN could not distinguish Cu and Pb categories well (Figure 24). The accuracy (*A*) and *F*-score were low, both less than 0.8 (Figure 25). Moreover, many of the precision and recall rates for the identification of Cu and Pb species in the modeling and *calibration* group were lower than 0.6 (Figure 25), which we did not want to observe. It can be seen that the use of the GRNN algorithm to distinguish Cu and Pb categories was unsuccessful. However, compared with the model established by FD and SD transformations, the Cu and Pb species could not be accurately distinguished.

It must be noted that the overall performance of the GRNN algorithm in distinguishing Cu and Pb species was poor and not as good as that of the SVM and ELM.

#### 3.3.4. Performance of the Method

The performances of the SVM and ELM algorithms were very similar for the distinction of Cu and Pb element types. The spectra were decomposed using MEMD, and the characteristic bands were screened using SPA, CARS, and IRIV. Finally, the categories of Cu and Pb were effectively identified and distinguished by the SVM and ELM algorithms. For the GRNN algorithm, there was no such good effect, and the types of Cu and Pb could not be accurately distinguished. Therefore, the performance of the GRNN algorithm was poor, which suggests that further spectral preprocessing may be required to achieve high classification accuracy. At the same time, the same classification algorithm based on FD and SD spectral transformation could not accurately distinguish the categories of Cu and Pb, or the classification and recognition effect of the *calibration* group was good, but the classification effect of the validation group was poor. There are two possible reasons for this. On the one hand, although the FD and SD transformations of the spectrum enhanced the correlation and highlighted the spectral features of the leaves, other details were ignored [58]. However, it also introduced noise, which reduced the signal-to-noise ratio [59]. This further proves the superiority of the model based on MEMD spectral transformation for distinguishing Cu and Pb element types.

### 3.4. Discussion

This study also provides a method that can visually distinguish and identify types of Cu and Pb elements, which further proves the model’s performance in determining Cu and Pb. In the above analysis, copper and lead pollution elements were used as representatives, combined with the machine learning algorithms, SVM, ELM, and GRNN, to build a model to distinguish the types of copper and lead elements. We drew a two-dimensional identification map to distinguish the types of Cu and Pb (Figure 8, Figure 10, Figure 12, Figure 14, Figure 16, Figure 18, Figure 20, Figure 22 and Figure 24). There were specific differences in the spectral responses of vegetation to different heavy metal stresses. However, this difference was sometimes very weak, which also lays the foundation of the model for effectively distinguishing and identifying copper and lead [60]. To fully exploit the weak differences in the leaf spectrum under heavy metal stress, signal spectrum analysis is considered to be a reliable method, which is also the reason for the introduction of MEMD transformation in this study [61].

The spectrum was decomposed into several IMF components by MEMD transformation, and components with better modal separation and feature retention were selected for research. Combined with the corresponding heavy metal content, SPA, CARS, and IRIV were used to screen the bands sensitive to the heavy metal copper and lead pollution. Finally, SVM, ELM, and GRNN were combined to build a model to distinguish the types of Cu and Pb. It can be seen from the results that by using the MEMD-SPA-SVM, MEMD-CARS-SVM, MEMD-SPA-ELM, MEMD-CARS-ELM, and MEMD-IRIV-ELM models, they could intuitively and effectively distinguish the spectra of maize leaves under copper and lead stress. The results obtained were reasonable. These satisfactory results indicate that it is feasible to use leaf spectra to distinguish the Cu and Pb elemental classes. Compared with other inversion models, these models have more advantages and no explicit requirements or restrictions on the data of the *calibration* and validation groups [62]. Among these three classification methods, SVM and ELM outperformed GRNN, partly because SVM and ELM are good at classification recognition of spectral data. In addition, compared with the FD and SD transformations of the spectrum, the components obtained after MEMD of the range can screen out more sensitive bands (Figure 7). This is mainly because MEMD decomposes the spectrum at the time and frequency scales, which can continuously decompose the spectrum to make it correspond to the original spectral data; thus, subtle spectral information can be more effectively identified from the original spectrum [63,64]. The MEMD spectral preprocessing method achieved satisfactory accuracy, except that the accuracy and *F*-score of the GRNN classification method in the identification of Cu and Pb was less than 0.8, and the classification and identification accuracy of SVM and ELM were both above 0.8. Therefore, when distinguishing the element types of Cu and Pb, the MEMD spectral transformation and combined SVM and ELM algorithms should be given priority. Screening the characteristic bands from the components obtained by MEMD spectral transformation can be used as an indicator to quickly distinguish the Cu and Pb element types, which helps to apply hyperspectral remote sensing technology to the detection and identification of large-scale environmental heavy metal pollution elements. Compared with traditional detection methods, the hyperspectral model proposed in this study had higher accuracy in distinguishing Cu and Pb pollution categories. Simultaneously, hyperspectral technology has a faster detection speed, lower cost, and avoids secondary environmental pollution. It is suitable for scientific research and production practices under certain precise conditions. Owing to the limitations of spectral data, increasing spectral diversity should be considered in future studies to identify various heavy metal pollution element species.

This study also demonstrated that spectral transformation for spectral data processing combined with machine learning is the most suitable method for distinguishing heavy metal types. At the same time, this conclusion also lays a foundation for identifying and monitoring heavy metal pollution element types in large-scale ecological environments in the wild. This study further revealed that maize leaves are more sensitive to heavy metal stress and can effectively monitor environmental heavy metal pollution [65]. The ultimate goal of this study is to move from indoor experiments to field monitoring and combine them with hyperspectral remote sensing images to achieve extensive area monitoring of environmental heavy metal pollution.

## 4. Conclusions

In general, we explored the identification of Cu and Pb element types. This is an effective method to detect and distinguish the types of Cu and Pb elements by using the spectral changes in the maize leaves under the stress of the heavy metals Cu and Pb. In this study, MEMD was introduced into hyperspectral data processing, and fundamental transformations of FD, SD, and MEMD were performed on the spectrum. SPA, CARS, and IRIV were used to filter the characteristic bands, reduce redundant data, and identify crucial spectral information. Finally, combined with SVM, ELM, and GRNN, models were built to distinguish the types of Cu and Pb. The results of this study demonstrated the following:

(1) Under Cu and Pb stress, the content of heavy metals in maize leaves increased with an increase in stress concentration, showing a significant positive correlation;

(2) MEMD of the spectrum can fully tap the weak information hidden in the spectral data;

(3) Three model precision indicators (i.e., accuracy (*A*), precision (*P*), recall (*R*)) and the total evaluation value *F*-score were used to evaluate model accuracy. The results showed that the MEMD-SPA-SVM, MEMD-CARS-SVM, MEMD-SPA-ELM, MEMD-CARS-ELM, and MEMD-IRIV-ELM models were the most accurate in distinguishing Cu and Pb species. These values were above 0.8, indicating a promising model. The MEMD-IRIV-SVM model was more suitable for determining Cu and Pb species;

In conclusion, the research results fully demonstrate the potential of MEMD transformation combined with the machine learning algorithms, SVM and ELM, in identifying the heavy metals copper and lead. Hyperspectral technology can rapidly and nondestructively detect and distinguish heavy metal pollution categories. This study provides a new method for identifying and differentiating heavy metal pollution elements in soil under vegetation cover in the wild.

This study was a preliminary exploration of the use of hyperspectral remote sensing technology to identify the types of heavy metal elements in maize leaves. Although this study achieved encouraging results in classifying heavy metal element species, the proposed method has limitations and requires improvement in future studies. This study was conducted only in an indoor pot experiment, and the variables were strictly controlled. In future research, in terms of experimental design, we will move from laboratory research to field planting, monitor the situation of heavy metal pollution during crop growth, and further explore the universality and stability of the proposed model.

## Data Availability

Data sharing is not applicable.
